# Imaging-based transformer model predicts early therapy response in advanced nasopharyngeal carcinoma: a dual-center study

**DOI:** 10.1186/s13244-025-02142-y

**Published:** 2025-12-02

**Authors:** Kexin Shi, Changlong Chen, Yinjiao Fei, Lei Qiu, Jinling Yuan, Yuchen Zhu, Jingyan Luo, Weilin Xu, Yuandong Cao, Shu Zhou

**Affiliations:** 1 Department of Radiation Oncology, The First Affiliated Hospital with Nanjing Medical University, Nanjing, China; 2 Department of Radiation Oncology, Sun Yat-sen Memorial Hospital, Sun Yat-sen University, Guangzhou, China

**Keywords:** Nasopharynx, Locally advanced nasopharyngeal carcinoma, Magnetic resonance imaging, Early response, Radiomics

## Abstract

**Introduction:**

Deep-learning methodologies for predicting early response in locally advanced nasopharyngeal carcinoma (LA-NPC) remain unvalidated, with techniques like 2.5-D imaging and Transformers underexplored.

**Materials and methods:**

MRI images from LA-NPC patients diagnosed between January 2020 and March 2024 at two centers were analyzed. Patients (*n* = 184) were split into training (*n* = 89), validation (*n* = 39), and test (*n* = 56) sets. Three segmentation models—SegResNet, Unet, and UnetR—automatically delineated regions of interest (ROIs). A 2.5D approach integrated adjacent tumor sections into a transfer learning framework, leading to three predictive models: Clinical, Transformer, and Combined. Performance was assessed using ROC curves, calibration curves, and decision curve analysis (DCA).

**Results:**

The Transformer model outperformed others, achieving AUCs of 0.968, 0.957, and 0.830 for the training, validation, and test sets, respectively. The Clinical model had lower AUCs (0.898, 0.759, 0.658). The Combined model, integrating clinical data, matched or exceeded Transformer performance, particularly in the test set (AUC = 0.874).

**Conclusion:**

The Combined model, leveraging Transformer architecture and clinical factors, demonstrates strong efficacy in predicting early response in LA-NPC patients undergoing chemoradiotherapy, suggesting its potential for improved personalized treatment.

**Critical relevance statement:**

This study critically validates a novel 2.5-D radiomic-Transformer fusion model that improves early response prediction for locally advanced nasopharyngeal carcinoma, directly advancing personalized chemoradiotherapy planning in clinical radiology.

**Key Points:**

- Early treatment response prediction in locally advanced nasopharyngeal carcinoma lacks validated deep learning models using 2.5D imaging and Transformers.
- Transformer-based model achieved superior predictive performance compared to clinical or combined models.
- Integrating clinical data with Transformer imaging analysis improves personalized chemoradiotherapy outcome prediction.

**Graphical Abstract:**

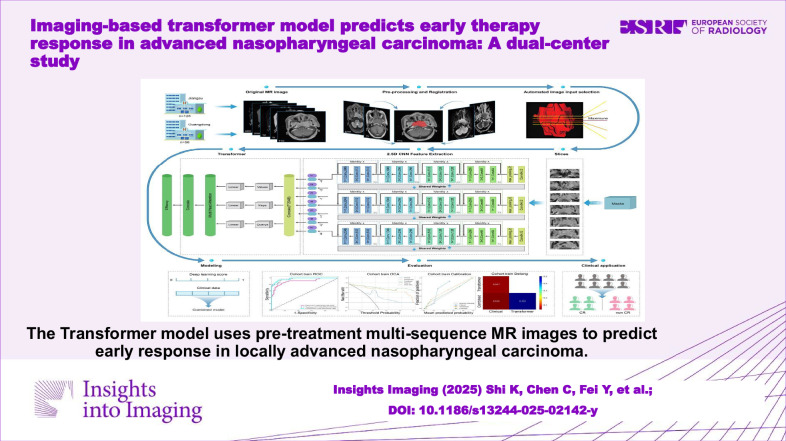

## Introduction

Nasopharyngeal carcinoma (NPC), a prevalent malignant tumor in China, exhibits significant regional clustering, with 80% of global cases occurring in various southern provinces of China [1–4]. Currently, the primary treatment for NPC is radiotherapy. 70% of patients are diagnosed at advanced stages (III and IVA) [5–9]. Early-stage (I–II) nasopharyngeal carcinoma shows a favorable prognosis post-treatment, with a 10-year overall survival rate ranging from 87.1 to 100%, whereas for stage III–IVA patients, it ranges from 55.5 to 75.6% [10]. Prognosis is poorer for LA-NPC patients with residual tumors compared to those achieving complete remission within 3 months post-radiochemotherapy [5, 11–14]. Previous studies have found that residual disease is a negative prognostic factor, contributing to poor survival [15, 16]. Therefore, a model providing recent therapeutic efficacy information upon nasopharyngeal carcinoma diagnosis would aid in treatment optimization and monitoring, significantly impacting individualized clinical management and patient survival rates.

MRI serves as a standard clinical tool for the diagnosis and staging of nasopharyngeal carcinoma [3, 17]. Although conventional hand-crafted radiomics based on MRI can be used to predict chemotherapy response, prognosis, and molecular subtypes, its application is constrained by the reliance on time-consuming and precise manual tumor annotation, which hinders widespread clinical adoption and efficient information retrieval [17–19]. Deep learning (a promising machine learning technique machine), overcomes these limitations by automatically processing raw data and recognizing tumor regions without manual feature extraction [20–24]. This approach has demonstrated performance comparable to experts across a range of medical image interpretation tasks [25–30]. Furthermore, there are currently no validated deep learning methods that can predict early responses in locally advanced nasopharyngeal carcinoma (LA-NPC). Additionally, advanced techniques such as 2.5D—a modeling approach that integrates both 2D and 3D elements to enhance feature extraction in medical Transformer by incorporating slice data alongside spatial context—and Transformer models have yet to be explored in this context.

In this study, we developed and validated a Transformer model based on Transformer methods using pre-treatment multi-sequence MR images to predict early response in LA-NPC. Additionally, our research includes automatic segmentation models for nasopharyngeal tumors and 2.5D Transformer slice segmentation, with CNNs used for feature extraction, which can further enhance the efficacy and generalization ability of predictive models. The use of dual-center data provides robust validation for the model.

## Materials and methods

### Ethics approval and consent to participate

This study adhered to the ethical standards of the Declaration of Helsinki. Ethical approval was provided by the Ethics Committee of the First Affiliated Hospital of Nanjing Medical University (Approval No. 2024-SR-609). Furthermore, data from patients with LA-NPC at the Second Affiliated Hospital of Sun Yat-sen University were included, with authorization obtained from its institutional ethics review board (Approval No. SYSKY-2024-613-01). Given the retrospective design and use of anonymized patient data, the requirement for informed consent was waived in accordance with institutional guidelines.

### Patients cohorts

We assembled two independent patient cohorts. The first cohort, originating from Nanjing, was divided into a training set and a validation set. This cohort included 289 patients with pathologically confirmed nasopharyngeal carcinoma, all treated at the First Affiliated Hospital of Nanjing Medical University between January 2020 and March 2024. Tumor staging followed the guidelines of the 8th edition of the American Joint Committee on Cancer (AJCC) TNM classification system [31].

**Fig. 1:**
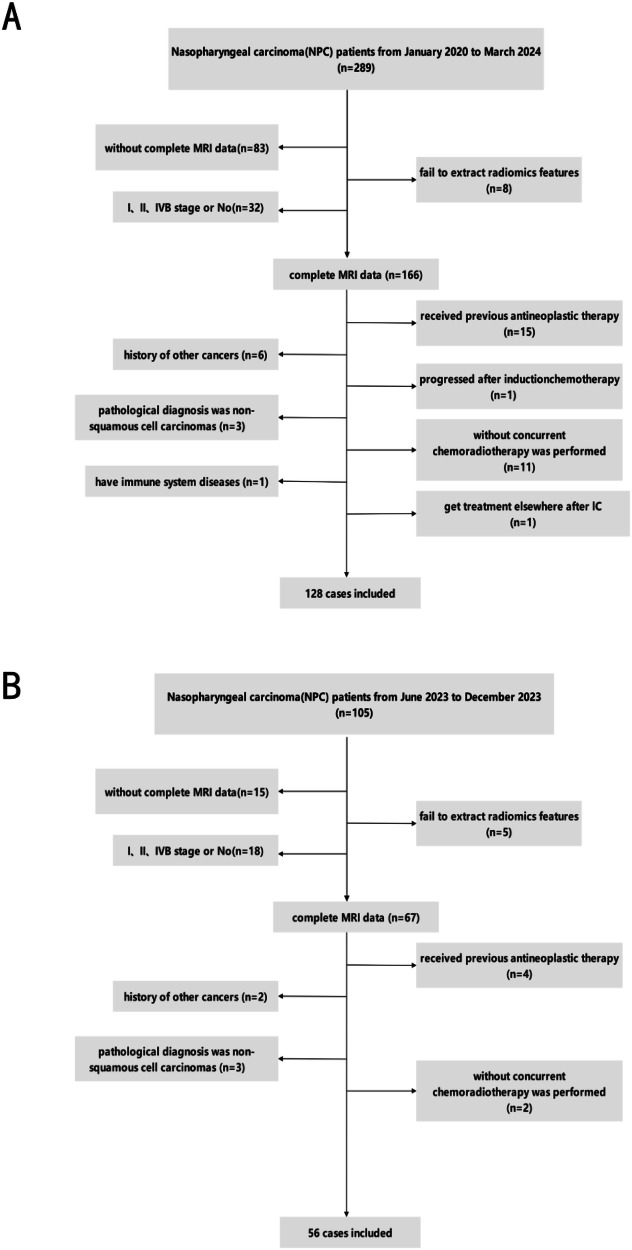
**A** Flowcharts illustrating the inclusion criteria for patients diagnosed with nasopharyngeal carcinoma, delineating the composition of both the training and validation sets. **B** Depicts the cohort of patients with nasopharyngeal carcinoma designated for the test set

Patients were retrospectively selected according to the following inclusion criteria: (1) a confirmed histological diagnosis of stage III–IVA squamous cell NPC, (2) completed a full course of radical CCRT, (3)available multiparametric MRI (including axial T1-WI, T1-C, T2-WI, DWI, and ADC) of the nasopharyngeal region acquired before and after treatment, (4) complete clinical data available, and (5) normal bone marrow, liver, and kidney function [15]. Exclusion criteria were: (1) MRI images with significant artifacts, poor clarity, or missing sequences, (2) history of other malignancies or prior treatment for nasopharyngeal carcinoma, and (3) long-term steroid use or immune-related diseases. Figure 1 provides a detailed overview of the patient selection process.

We collected data for an external test cohort from the Second Affiliated Hospital of Sun Yat-sen University, which included 105 patients with pathologically confirmed nasopharyngeal carcinoma between June and December 2023. The inclusion and exclusion criteria applied to this cohort were identical to those used for the primary cohort, as detailed in Fig. 1.

### MRI image acquisition and segmentation

**Fig. 2:**
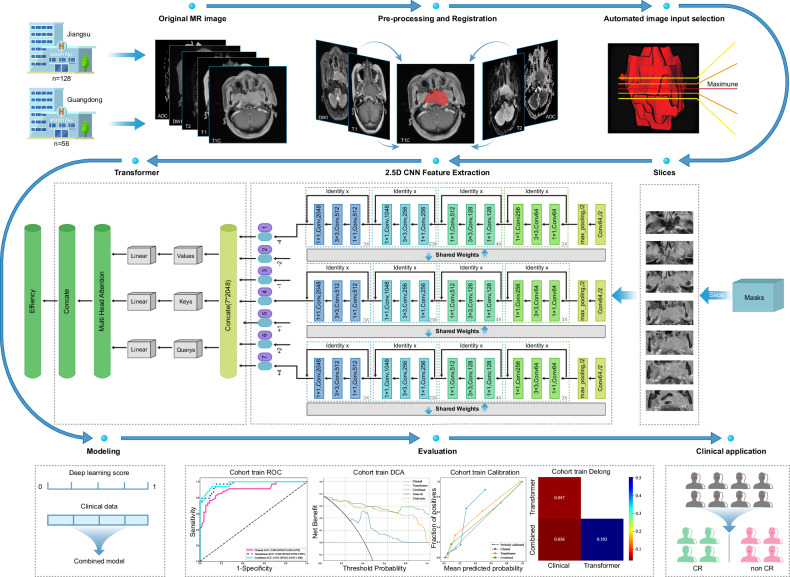
Overview of the workflow of this study

Figure 2 shows the entire workflow of our study. Imaging for all patients was performed using 3.0 T MRI scanners. DICOM format images of axial T1WI, T1-C, T2WI, DWI, and ADC were retrieved for each case using PACS (Carestream, Ontario, Canada). In the ITK-SNAP software, all regions of interest (ROIs) were independently manually delineated by two radiation oncologists with 3-year and 8-year NPC experience, based on T1-CE MRI (the gold standard), which provides the clearest anatomical structure. The contours underwent registration, discrepancy comparison, and consensus discussion, achieving high inter-rater agreement (Cohen’s Kappa κ = 0.86 > 0.8, indicating nearly complete agreement between the two radiotherapists, see details in Table S1). The remaining four sequences were registered to the T1-CE space, with segmentation results uniformly calibrated by a senior expert (8 years’ experience) [15].

### Automated segmentation and ROI processing

We randomly divided eligible patients from the primary cohort into a training set (*n* = 89) and a validation set (*n* = 39) at a ratio of 7:3. To significantly enhance automation in medical image analysis, we employed the SegResNet, Unet, and UnetR models for the automatic segmentation of regions of interest (ROIs), thereby minimizing the need for manual intervention and increasing operational efficiency. To validate the performance of the automated segmentation algorithm in subsequent modeling [32]. The model with the highest Dice coefficient on the validation set (SegResNet; see Table S4) was selected for all subsequent analyses. The trained SegResNet model was used to automatically generate 3D tumor masks, from which the ROIs were cropped.

### 2.5D-transformer feature fusion

We applied 2.5D technology to extract adjacent slices in the up-down and anterior-posterior directions from the selected center slice. Offsets of ± 1, ± 2, and ± 4 were applied, yielding a total of seven 2D slices per patient centered on the maximum cross-section of the ROI, referred to as a 2.5D dataset. By analyzing these 2.5D datasets through transfer learning with various pre-trained models (including DenseNet121, ResNet50, ResNet101, VGG19) and InceptionV3 [33], we effectively extracted and utilized deep learning features. Additionally, our approach incorporates a Transformer-based multi-instance learning framework that synthesizes individual slice features into a unified prediction model, thereby enhancing analytical capabilities and overall accuracy in medical diagnostics.

### Clinical feature selection and model fusion

Clinical features were analyzed to identify predictors for model integration. We first performed univariate analysis; variables with a *p*-value < 0.05 were then entered into multivariate analysis. Significance in the multivariate model (*p* < 0.05) served as the criterion for final selection. To build the Combined model, the selected clinical feature (tumor_volume) and the deep learning-derived Transformer_Feature were first standardized (Z-score normalization) and then concatenated. This combined feature vector was used to train a logistic regression classifier. The resulting model coefficients are reported in the “Results” section. For comparison, a pure Clinical model was built using an ExtraTrees classifier, chosen for its proficiency in modeling non-linear interactions, trained solely on the selected clinical feature (tumor_volume). Selection procedure in Table S3.

### Outcome definition and statistical analysis

Treatment response was assessed on 3-month post-CCRT MRI by two radiologists according to RECIST 1.1 guidelines [34]. Patients were classified as either complete response (CR) or non-CR (grouping partial response (PR), stable disease (SR), and progressive disease (PD)). An excellent inter-rater agreement was achieved (Cohen’s κ = 0.93; see Table S2). Model performance was evaluated by the area under the receiver operating characteristic curve (AUC). The Hosmer-Lemeshow test and calibration curves assessed model calibration. Decision curve analysis (DCA) evaluated clinical utility.

We employed the Shapiro–Wilk test to assess the normality of clinical features. For continuous features with normal distribution, we applied *t*-tests, and for those without normal distribution, we used the Mann–Whitney U test. We evaluated categorical variables using the chi-square (χ²) test. We performed all statistical analyses and developed the machine learning models in Python (version 3.7.12) and scikit-learn (version 1.0.2). We conducted the entire training process on an NVIDIA 4090 GPU with MONAI 0.8.1 and PyTorch 1.8.1. We considered all statistical tests two-sided and regarded *p* < 0.05 as statistically significant.

## Results

### Patient demographic characteristics

In the main cohort, we included a total of 128 patients, 89 in the training set, and 39 in the internal validation set. For the external validation set, a total of 56 patients were enrolled. Table 1 indicates that no statistical differences in baseline information between the training and validation sets were observed (*p* > 0.05), ensuring the comparability for subsequent modeling and validation.

**Table 1:** Characteristics of patients in training, validation, and test sets

	Train cohort			Val cohort			Test cohort		
feature_name	CR	Non-CR	*p*-value	CR	Non-CR	*p*-value	CR	Non-CR	*p*-value
Age	53.33 ± 11.28	51.94 ± 13.12	0.598	54.07 ± 12.81	53.00 ± 12.69	0.964	45.83 ± 11.99	44.37 ± 13.81	0.674
Height	165.33 ± 7.16	169.06 ± 7.63	0.022	168.19 ± 6.24	164.67 ± 5.84	0.106	162.28 ± 8.74	165.04 ± 7.97	0.223
Weight	66.19 ± 12.33	68.09 ± 14.44	0.557	66.31 ± 10.32	64.29 ± 11.77	0.592	59.95 ± 11.87	67.67 ± 15.19	0.038
BMI	23.99 ± 3.55	23.69 ± 3.72	0.708	23.36 ± 2.97	23.62 ± 3.60	0.819	22.64 ± 3.29	24.77 ± 4.95	0.061
WBC	6.80 ± 2.05	6.93 ± 2.08	0.883	6.84 ± 2.04	6.30 ± 1.55	0.426	6.83 ± 1.57	7.71 ± 1.56	0.027
Lymphocyte	1.58 ± 0.49	1.49 ± 0.43	0.379	1.60 ± 0.52	1.46 ± 0.60	0.455	1.84 ± 0.62	1.79 ± 0.55	0.774
Monocyte	0.49 ± 0.20	0.50 ± 0.21	0.849	0.46 ± 0.16	0.46 ± 0.18	0.944	0.51 ± 0.23	0.60 ± 0.18	0.013
Neutrophils	4.59 ± 2.04	4.76 ± 1.85	0.651	4.62 ± 1.77	4.19 ± 1.21	0.659	4.27 ± 1.21	5.06 ± 1.25	0.019
PLT	244.60 ± 87.63	254.12 ± 85.15	0.317	225.85 ± 49.77	266.33 ± 104.78	0.377	268.31 ± 66.38	273.04 ± 69.11	0.795
NLR	3.47 ± 2.69	3.80 ± 3.36	0.364	3.20 ± 1.67	3.45 ± 2.03	0.749	2.84 ± 2.54	3.19 ± 1.56	0.091
MLR	0.33 ± 0.14	0.36 ± 0.25	0.615	0.32 ± 0.16	0.35 ± 0.19	0.738	0.32 ± 0.24	0.36 ± 0.17	0.028
PLR	170.98 ± 87.17	196.72 ± 139.55	0.415	152.10 ± 48.56	204.48 ± 85.97	0.066	172.82 ± 107.29	171.07 ± 86.51	0.658
ALP	84.62 ± 21.29	87.74 ± 27.22	0.853	83.07 ± 17.85	85.33 ± 22.02	0.736	72.90 ± 18.18	83.11 ± 24.22	0.086
LDH	198.36 ± 46.11	207.76 ± 73.12	0.807	192.19 ± 42.76	206.17 ± 47.57	0.368	184.86 ± 25.73	218.56 ± 52.32	0.002
Alb_g_L	41.51 ± 3.72	41.59 ± 4.10	0.922	41.29 ± 3.30	39.77 ± 2.51	0.164	42.49 ± 3.28	43.06 ± 3.30	0.515
D_Dimer	0.46 ± 0.65	0.42 ± 0.43	0.839	0.59 ± 1.29	0.93 ± 1.54	0.185	0.30 ± 0.16	0.64 ± 1.08	0.253
tumor_volume	48.17 ± 26.63	73.56 ± 60.31	0.106	55.72 ± 33.61	92.07 ± 55.76	0.063	45.36 ± 24.32	37.06 ± 39.78	0.023
Gender			0.101			1.0			0.5
0	16 (29.09)	4 (11.76)		7 (25.93)	3 (25.00)		11 (37.93)	7 (25.93)	
1	39 (70.91)	30 (88.24)		20 (74.07)	9 (75.00)		18 (62.07)	20 (74.07)	
Smoking			0.845			1.0			0.636
0	38 (69.09)	22 (64.71)		20 (74.07)	9 (75.00)		25 (86.21)	21 (77.78)	
1	17 (30.91)	12 (35.29)		7 (25.93)	3 (25.00)		4 (13.79)	6 (22.22)	
Drinking			1.0			0.258			0.971
0	41 (74.55)	25 (73.53)		22 (81.48)	7 (58.33)		29 (100.00)	26 (96.30)	
1	14 (25.45)	9 (26.47)		5 (18.52)	5 (41.67)		null	1 (3.70)	
Family_history_of_NPC			0.856			1.0			1.0
0	52 (94.55)	31 (91.18)		27 (100.00)	12 (100.00)		28 (96.55)	26 (96.30)	
1	3 (5.45)	3 (8.82)		null	null		1 (3.45)	1 (3.70)	
EBV_DNA			1.0			0.559			< 0.001
0	31 (56.36)	19 (55.88)		16 (59.26)	9 (75.00)		null	13 (48.15)	
1	24 (43.64)	15 (44.12)		11 (40.74)	3 (25.00)		29 (100.00)	14 (51.85)	
T_stage			0.377			0.452			0.055
1	5 (9.09)	1 (2.94)		1 (3.70)	null		3 (10.34)	1 (3.70)	
2	12 (21.82)	4 (11.76)		1 (3.70)	null		15 (51.72)	6 (22.22)	
3	18 (32.73)	14 (41.18)		16 (59.26)	5 (41.67)		8 (27.59)	13 (48.15)	
4	20 (36.36)	15 (44.12)		9 (33.33)	7 (58.33)		3 (10.34)	7 (25.93)	
Lymph_node_metastasis			0.674			0.323			0.7
0	1 (1.82)	null		null	null		null	null	
1	14 (25.45)	7 (20.59)		11 (40.74)	2 (16.67)		4 (13.79)	2 (7.41)	
2	23 (41.82)	18 (52.94)		12 (44.44)	8 (66.67)		19 (65.52)	20 (74.07)	
3	17 (30.91)	9 (26.47)		4 (14.81)	2 (16.67)		6 (20.69)	5 (18.52)	
Clinical_stage			1.0			0.35			1.0
1	20 (36.36)	13 (38.24)		15 (55.56)	4 (33.33)		19 (65.52)	17 (62.96)	
2	35 (63.64)	21 (61.76)		12 (44.44)	8 (66.67)		10 (34.48)	10 (37.04)	
Cycle_of_Induction_chemotherapy			0.697			0.934			0.766
0	23 (41.82)	12 (35.29)		11 (40.74)	4 (33.33)		14 (48.28)	11 (40.74)	
1	32 (58.18)	22 (64.71)		16 (59.26)	8 (66.67)		15 (51.72)	16 (59.26)	

### Automatic segmentation performance

Among the three evaluated segmentation architectures (UNETR, Unet, SegResNet), SegResNet demonstrated the highest Dice coefficients (0.857 in training; 0.866 in validation, Table S4) and was therefore selected to generate all subsequent tumor masks for feature extraction.

### Predictor selection and model development

Univariate analysis of clinical features identified tumor volume (OR: 1.147, 95% CI: 1.055–1.247, *p* = 0.008) and height (OR: 1.126, 95% CI: 1.035–1.225, *p* = 0.022) as significant predictors (*p* < 0.05). Following multivariate analysis refinement, tumor volume (OR: 1.124, 95% CI: 1.033–1.223, *p* = 0.024) emerged as the sole significant clinical predictor. Consequently, tumor volume was selected as the only clinical feature for inclusion in the comprehensive model and ExtraTrees classifier development. Complete statistical results are presented in Table S5 and Fig. S2.

The final combined model integrated the deep learning-derived image feature (Transformer_Feature) with the significant clinical predictor (tumor_volume) by concatenating the standardized features and inputting them into a logistic regression classifier. The resulting formula was: Combined_Score = −0.039 × tumor_volume_std + 15.914 × Transformer_Feature_std - 3.491. A nomogram visualizing this model is presented in Fig. S4.

### Comparative performance of predictive models

**Fig. 3:**
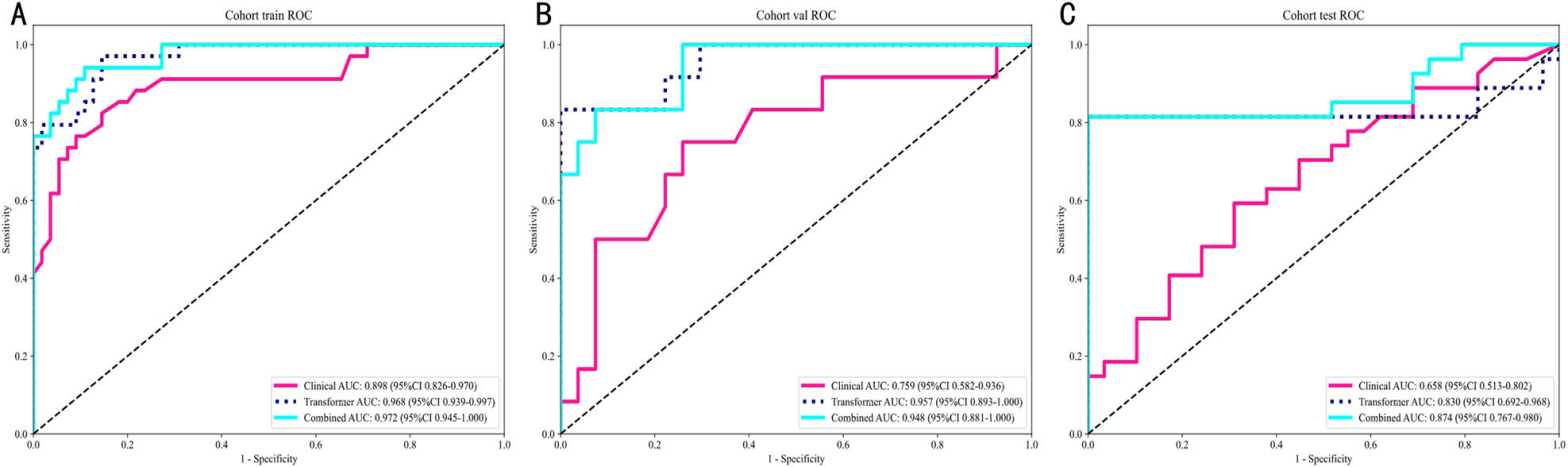
Different signatures’ ROC on different cohorts. **A** Cohort train ROC (Clinical AUC: 0.898; Transformer AUC: 0.968; Combined AUC: 0.972); **B** Cohort val ROC (Clinical AUC: 0.759; Transformer AUC: 0.957; Combined AUC: 0.948); **C** Cohort val ROC (Clinical AUC: 0.658; Transformer AUC: 0.830; Combined AUC: 0.874)

Table 2 compares three predictive signatures across cohorts using AUC metrics. The Transformer model achieved superior AUCs (training: 0.968; validation: 0.957), demonstrating high reliability. It maintained robustness in testing (AUC = 0.830), outperforming the Clinical signature. The Combined signature exceeded Transformer performance in testing (AUC = 0.874), highlighting the value of clinical-deep learning integration. ROC curves are shown in Fig. 3.

**Table 2:** Metrics on different signatures

Signature	Accuracy	AUC	95% CI	Sensitivity	Specificity	PPV	NPV	Cohort
Clinical	0.831	0.898	0.8259–0.9704	0.794	0.855	0.771	0.870	Train
Transformer	0.888	0.968	0.9386–0.9972	0.941	0.855	0.800	0.959	Train
Combined	0.899	0.972	0.9448–0.9996	0.912	0.891	0.838	0.942	Train
Clinical	0.718	0.759	0.5823–0.9362	0.667	0.741	0.533	0.833	val
Transformer	0.923	0.957	0.8931–1.0000	0.750	1.000	1.000	0.900	val
Combined	0.872	0.948	0.8808–1.0000	0.750	0.926	0.818	0.893	val
Clinical	0.625	0.658	0.5132–0.8022	0.556	0.690	0.625	0.625	test
Transformer	0.893	0.830	0.6920–0.9683	0.778	1.000	1.000	0.829	test
Combined	0.893	0.874	0.7667–0.9804	0.778	1.000	1.000	0.829	test

**Fig. 4:**
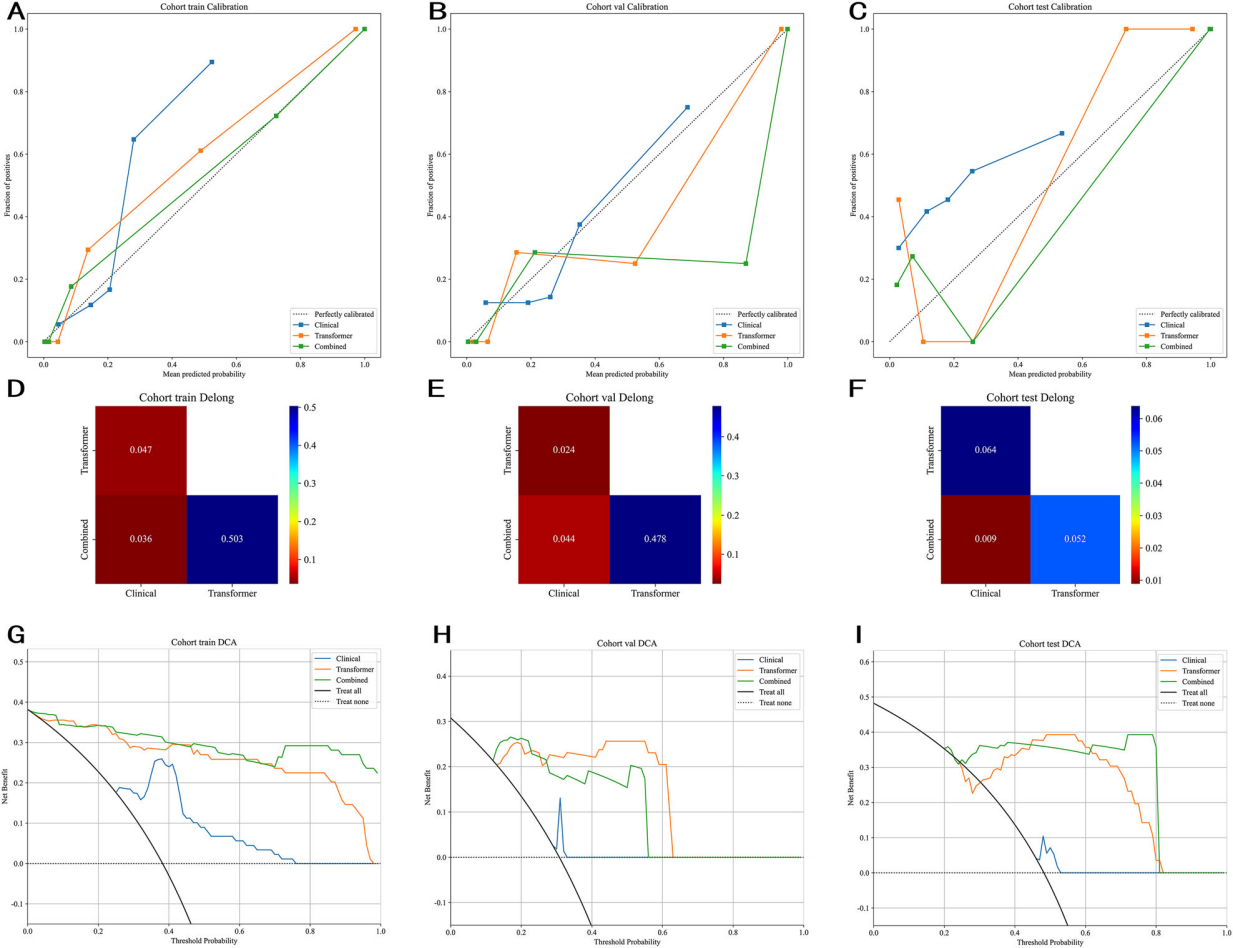
Signature comparison on all cohorts. **A** Calibration curves in train cohort; **B** Calibration curves in val cohort; **C** Calibration curves in test cohort; **D** Delong test in train cohort; **E** Delong test in val cohort; **F** Delong test in val cohort; **G** Cohort train DCA; **H** Cohort val DCA; **I** Cohort test DCA

We used the Hosmer-Lemeshow (HL) test to evaluate how well model predictions matched actual outcomes. A lower HL statistic indicates better alignment. As shown in Fig. 4, our Combined model (clinical + deep learning features) achieved the best calibration (HL *p* > 0.05). Across training and test datasets, the Combined model outperformed single-feature models. It showed noticeable improvement over the Clinical model alone (Delong test *p* < 0.05). Decision curve analysis (DCA) in Fig. 4 further confirmed that the Combined model provides greater practical benefit—delivering higher net gains across different prediction probabilities compared to other approaches.

### Model interpretability

To enhance the interpretability of deep learning-based image features, we employ Gradient CAM. This method generates heat maps that highlight regions critical to model predictions. By visually comparing these heat maps with original input images, we can precisely identify and interpret key anatomical features driving classification decisions. Despite the model’s complex architecture, its rational feature activation patterns (Fig. S3) still strengthen confidence in core model regions.

## Discussion

We developed a multi-instance combined learning model based on a Transformer architecture. This approach integrates 2.5D Transformer techniques with automatic segmentation to generate diagnostic slices, followed by CNN-based feature extraction for predicting early treatment response in patients with locally advanced nasopharyngeal carcinoma (LA-NPC).

Patients with locally advanced nasopharyngeal carcinoma (LA-NPC) who fail to achieve a complete response (non-CR) following chemoradiotherapy are known to have significantly poorer survival outcomes compared to CR achievers [5, 13, 14, 35–37]. The accurate and early prediction of early treatment response is therefore critically important for risk stratification and subsequent therapeutic decision-making. As demonstrated by Cao et al [38], patients with non-CR may benefit from intensified treatment regimens—such as trilaciclib combined with capecitabine—which can significantly improve survival. This underscores the vital role of reliable CR prediction in guiding timely and personalized treatment interventions. Furthermore, the NCCN 2025 Guidelines explicitly recommend that CR assessment at 4–8 weeks post-radiotherapy should serve as a major clinical decision point. Patients with residual lesions require prompt enhanced imaging and aggressive treatment, including potential salvage surgery or clinical trials with novel agents, while CR patients can safely continue with less intensive monitoring. Thus, the development of robust predictive models for early CR is not only methodologically significant but also essential for enabling stratified patient management, optimizing resource allocation, and ultimately improving survival in LA-NPC.

Current prognostic feature extraction from medical images faces dual challenges: traditional radiomics requires labor-intensive tumor delineation despite capturing intratumoral heterogeneity, while deep learning struggles to isolate tumor-specific signals from whole-image analysis. Our approach resolves this by combining automatic tumor segmentation with transfer learning-enhanced 2.5D feature extraction from masked ROIs.

We replaced manual tumor outlining with automated segmentation to improve efficiency and consistency. Three models were tested using standard Dice scores. This automated method generates tumor delineations more rapidly and reliably than manual approaches, thereby improving the consistency of our AI-based early treatment response prediction. These improvements directly help doctors plan treatments and care for nasopharyngeal cancer patients [20, 39].

Prior studies relied on conventional 2D deep learning methods [26, 40], which often fail to capture sufficient spatial context. To overcome this limitation, we implemented a 2.5D approach that integrates adjacent NPC MRI slices, enhancing spatial information utilization and model generalizability. By training on orthogonal planes (axial, sagittal, coronal) while preserving 2D network efficiency [41], our method significantly improves segmentation performance, reproducibility, and scalability compared to traditional 2D frameworks. This research focuses on developing a Combined model based on Transformers and employs a multi-instance learning approach to integrate results from seven slices representing each patient, surpassing traditional average probability methods. This method uniquely confirms each slice as a single instance. Given the diversity of models used in our 2.5D deep learning framework, we selectively merged results from the best-performing model into the validation set. Transformers, as a deep learning model, have emerged as an alternative to CNNs in non-medical computer vision tasks [42–44]. To our knowledge, despite their superior performance compared to CNNs, Transformers have not been widely applied for early treatment response prediction in nasopharyngeal carcinoma [45].

Our research group previously predicted early response in LA-NPC using radiomics [15]. Internally validated in a single-center environment. The Combined model (integrating clinical and Transformer factors) demonstrated superior performance to the pure clinical model. In contrast, our Transformer-based model not only demonstrated superior performance but also underwent validation across larger sample sizes and different centers. In this study, after integrating clinical factors validated to correlate with prognosis into the Transformer model, the AUC improved by 0.07, consistent with some previous studies. The strategy of combining multi-task deep learning and radiomics has been used for prognosis prediction in head and neck cancer, achieving among the highest prognostic performance in the HECKTOR 2022 challenge for segmentation and outcome prediction of head and neck tumors. Our research further validates this strategy. Identifying patients with high-risk non-CR remains challenging. Our deep learning model could help identify high-risk patients who may benefit from more aggressive treatment.

This study has several limitations. First, the retrospective design combined with a relatively small sample size carries inherent risks of selection bias and unmeasured confounders, and limited the statistical power for subgroup analyses—particularly for patients with partial response/stable disease (PR/SD). To address this, we will expand our cohort through multicenter collaborations, which will enable robust validation of PR/SD subgroup characteristics and facilitate the exploration of personalized therapeutic strategies. Second, the absence of long-term follow-up data precludes assessment of the model’s predictive durability. Future prospective studies will track sustained outcomes to determine whether early response forecasts correlate with long-term survival. Third, exclusive reliance on MRI and the lack of histopathological verification constrain biological comprehensiveness and impede imaging–pathology correlation analysis. We plan to integrate complementary modalities (e.g., PET-CT radiomics) and pursue histopathological sampling in subsequent studies to enhance biological interpretability and prediction robustness.

In conclusion, we developed and validated a robust, automated deep learning model for accurate early treatment response prediction in LA-NPC patients across two independent centers. By integrating automated 2.5D segmentation with a transformer-based multi-instance learning framework, our approach effectively overcomes key limitations of conventional radiomics and deep learning methods. Following successful prospective validation, this model holds strong potential as a clinical decision-support tool, enabling optimized post-treatment surveillance and facilitating timely interventions for high-risk patients to improve overall outcomes.

## Supplementary information

Supplementary information

## Data Availability

Due to the retrospective design of this study and the absence of patient consent for public data release, the raw data underlying this article’s conclusions cannot be made publicly available. The data include potentially sensitive personal information that could risk compromising participant privacy. However, data access may be granted by the corresponding author upon reasonable request, contingent on approval from the relevant institutional review boards. All data requests will be carefully reviewed to ensure compliance with the ethical guidelines and privacy commitments established during the consent process.

## Abbreviations

ADC: Apparent diffusion coefficient
AJCC: American Joint Committee on Cancer
AUC: Area under the curve
CCRT: Completion of radical concurrent chemoradiotherapy
CNNs: Convolutional neural networks
CR: Complete response
DCA: Decision curve analysis
DWI: Diffusion-weighted
HL: Hosmer-Lemeshow
LA-NPC: Locally advanced nasopharyngeal carcinoma
NPC: Nasopharyngeal carcinoma
OR: Odds ratio
PD: Progressive disease
PR: Partial response
ROIs: Regions of interest
SD: Stable disease
