# Supplementary material for: Imaging-based transformer model predicts early therapy response in advanced nasopharyngeal carcinoma: a dual-center study
Source: Insights Imaging. 2025 Dec 2;16:267. doi: 10.1186/s13244-025-02142-y (PMC12673000; doi:10.1186/s13244-025-02142-y)

Imaging-Based Transformer Model Predicts Early Therapy Response in Advanced Nasopharyngeal Carcinoma: A Dual-Center Study

ELECTRONIC SUPPLEMENTARY MATERIAL

Title	Page
Table S1. Consistency of Handcraft tumor segmentation	2
Table S2. Consistency of efficacy evaluation	2
Table S3. Metric results for Clinical Signature	3
Table S4: Comparison of different segmentation models	4
Table S5: Univariable and Multivariable Analysis of clinical features	5-6
Figure S1: Depicts the architecture of the SegResNet network	7
Figure S2: a. OR of clinical features in univariable analysis.b. OR of clinical features in multivariable analysis	8
Figure S3: Examples of gradient-weighted class activation mappings (Grad-CAMs)	9
Figure S4: Fused Clinical-Radiomic Nomogram	10

Table S1.Consistency of Handcraft tumor segmentation

	Observer B: Positive	Observer B: Negative
Observer A: Positive	93	12
Observer A: Negative	1	78

k=0.86>0.8 indicates almost perfect agreement.

Table S2. Consistency of efficacy evaluation

	Observer B: CR	Observer B: non-CR
Observer A: CR	108	3
Observer A: non-CR	3	70

k=0.93>0.8 indicates almost perfect agreement.

Table S3. Metric results for Clinical Signature

model_name	Accuracy	AUC	95% CI	Sensitivity	Specificity	PPV	NPV	Cohort
LR	0.708	0.756	0.654 - 0.858	0.412	0.891	0.700	0.710	train
LR	0.718	0.670	0.479 - 0.861	0.417	0.852	0.556	0.767	val
LR	0.607	0.599	0.445 - 0.753	0.407	0.793	0.647	0.590	test
ExtraTrees	0.708	0.898	0.826 - 0.970	0.235	1.000	1.000	0.679	train
ExtraTrees	0.718	0.759	0.582 - 0.936	0.167	0.963	0.667	0.722	val
ExtraTrees	0.589	0.658	0.513 - 0.802	0.148	1.000	1.000	0.558	test
XGBoost	0.775	0.880	0.810 - 0.950	0.471	0.964	0.889	0.746	train
XGBoost	0.667	0.656	0.453 - 0.859	0.417	0.778	0.455	0.750	val
XGBoost	0.589	0.642	0.497 - 0.788	0.296	0.862	0.667	0.568	test

Table S4: Comparison of different segmentation models

model	Dice	mIOU	SA	OS	US	cohort
Unetr	0.788	0.708	0.766	0.068	0.196	Train
Unetr	0.777	0.698	0.742	0.058	0.222	Val
Unet	0.739	0.668	0.720	0.084	0.225	Train
Unet	0.779	0.698	0.758	0.073	0.204	Val
Segresnet	0.857	0.783	0.871	0.074	0.105	Train
Segresnet	0.866	0.793	0.876	0.070	0.103	Val

Table S5: Univariable and Multivariable Analysis of clinical features

feature_name	OR	OR lower 95% CI	OR upper 95% CI	p_value	OR	OR lower 95% CI	OR upper 95% CI	p_value
Lymphocyte	0.955	0.875	1.042	0.379				
Age	0.973	0.891	1.061	0.598				
BMI	0.980	0.899	1.069	0.708				
D_Dimer	0.983	0.901	1.073	0.743				
Clinical_stage	0.991	0.908	1.081	0.861				
EBV_DNA	1.002	0.919	1.093	0.965				
Alb_g_L	1.005	0.921	1.096	0.922				
Drinking	1.006	0.921	1.097	0.916				
Monocyte	1.010	0.926	1.102	0.849				
Lymph_node_metastasis	1.013	0.929	1.105	0.808				
WBC	1.015	0.931	1.107	0.778				
Neutrophils	1.021	0.935	1.113	0.700				
Smoking	1.022	0.937	1.115	0.672				
PLT	1.027	0.941	1.120	0.616				
NLR	1.027	0.942	1.121	0.608				
ALP	1.032	0.946	1.125	0.548				
Cycle_of_Induction_ch	1.0	0.94	1.12	0.54				

Table S5: Univariable and Multivariable Analysis of clinical features

feature_name	OR	OR lower 95% CI	OR upper 95% CI	p_value	OR	OR lower 95% CI	OR upper 95% CI	p_value
emotherapy	32	6	6	6				
Family_history_of_NPC	1.032	0.946	1.126	0.543				
Weight	1.035	0.949	1.129	0.511				
LDH	1.040	0.953	1.134	0.458				
MLR	1.048	0.961	1.142	0.375				
PLR	1.057	0.969	1.153	0.287				
T_stage	1.081	0.992	1.178	0.134				
Gender	1.104	1.013	1.202	0.058				
Height	1.126	1.035	1.225	0.022	1.097	1.008	1.194	0.072
tumor_volume	1.147	1.055	1.247	0.008	1.124	1.033	1.223	0.024

Figure S1: depicts the architecture of the SegResNet network.

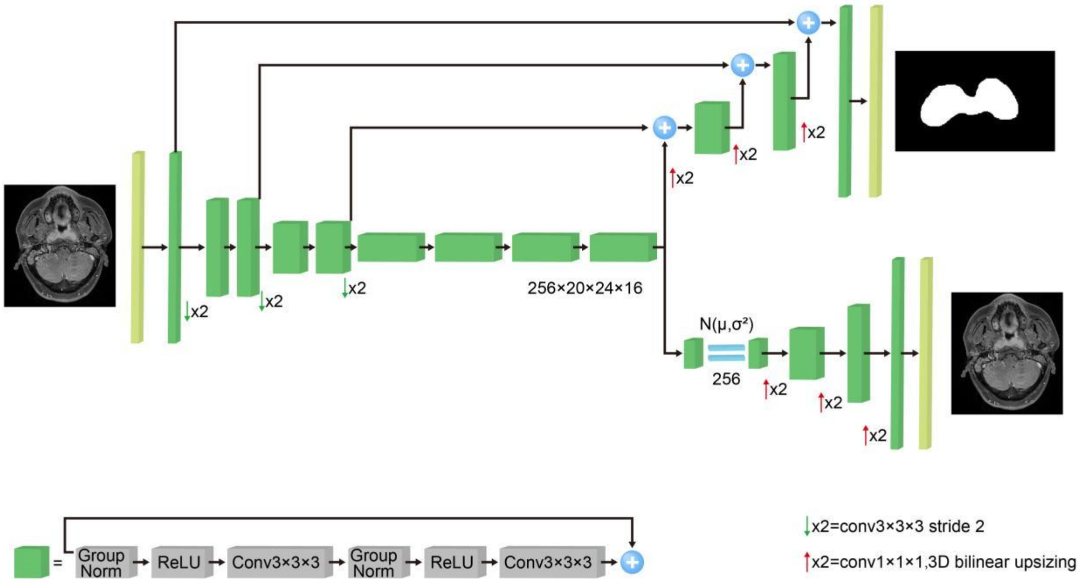

Figure S2: a. OR of clinical features in univariable analysis.b. OR of clinical features in multivariable analysis

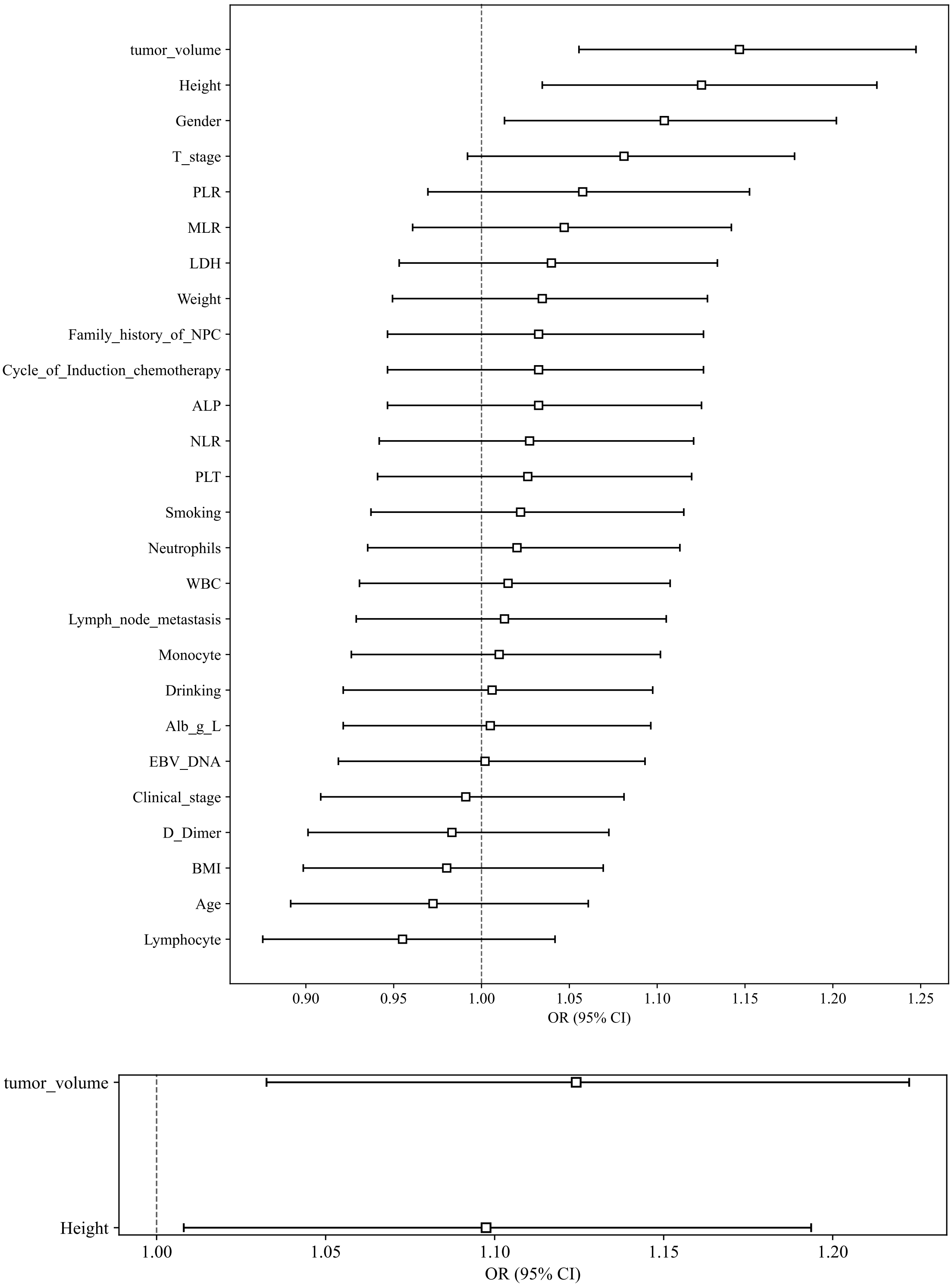

Figure S3: Examples of gradient-weighted class activation mappings (Grad-CAMs)

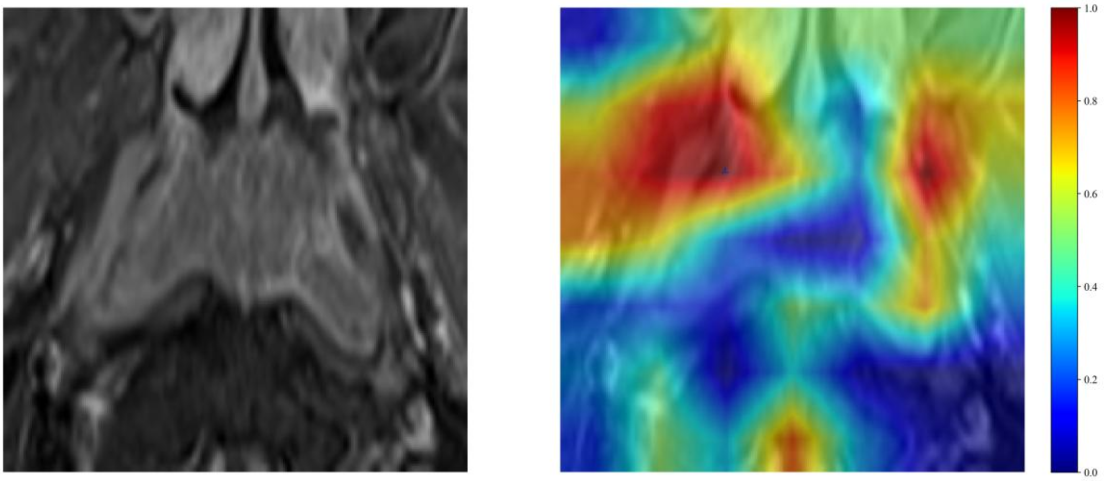

Figure S4: Fused Clinical-Radiomic Nomogram

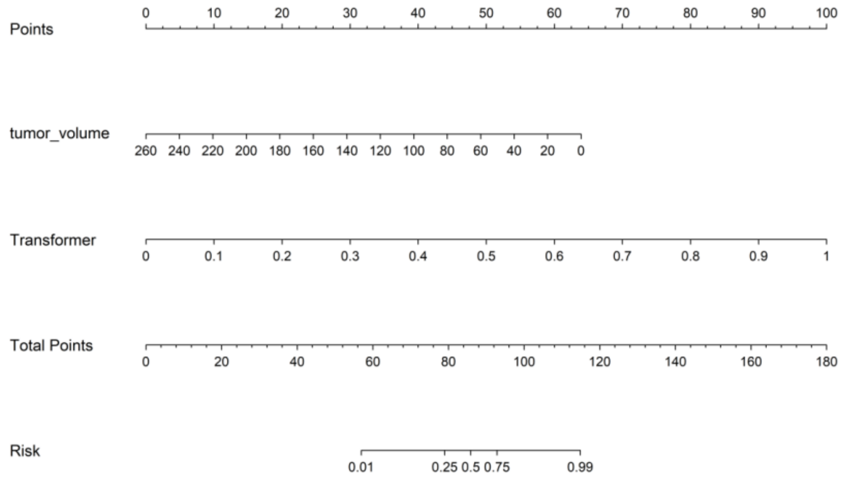

Supplement: Supplementary file 1 — Supplementary information [file 13244_2025_2142_MOESM1_ESM.pdf]
